# Testing for hereditary cancer genes in men: a missed opportunity for cancer prevention

**DOI:** 10.3389/fonc.2026.1766711

**Published:** 2026-03-16

**Authors:** Calan Szmyd, Madison LaFleur, Ashley Cantu-Weinstein, Jingwen Zhang, Nicholas Sun, Youbao Sha, Jessica L. Saben, Adam C. ElNaggar, Minetta C. Liu, Sheetal Parmar, Vivienne Souter

**Affiliations:** Natera, Inc., Austin, TX, United States

**Keywords:** BRCA, cancer prevention, cancer risk, germline genetic testing, hereditary cancer testing, men’s health

## Abstract

**Introduction:**

Genetic testing for inherited cancer risk syndromes can inform targeted surveillance and intervention, but testing for male patients is inadequately addressed by medical literature and clinical guidelines despite having higher cancer-related mortality and morbidity than women. Here, we evaluate characteristics and hereditary cancer testing practices and outcomes in men compared to women.

**Methods:**

This retrospective study analyzed hereditary cancer testing data from a commercial laboratory between 06/2020–08/2023. Outcomes assessed included demographics, personal and family cancer histories, ordering clinic type, gene panel size, test results (pathogenic/likely pathogenic, or negative), and the frequency of clinically actionable variants based on National Comprehensive Cancer Network and other guidelines. Comparisons between men and women were analyzed using odds ratios from Fisher’s exact test and Wilcoxon rank test.

**Results:**

Of the 224,041 individuals receiving hereditary cancer testing, only 10,936 (5%) were men. Compared to women, men were significantly older at testing (54 *vs*. 43 years; p<0.001), had higher odds of personal cancer history (OR, 2.67; 95% CI, 2.55 to 2.79), and had lower odds of family cancer history (OR, 0.50; 95% CI, 0.48 to 0.53). Testing orders for men were more likely to come from primary care or specialty clinics. Men had higher odds of testing positive for a pathogenic or likely pathogenic variant and for actionable gene variants.

**Discussion:**

Men represented a minority in genetic testing for hereditary cancers but had higher test positivity rates. These findings underscore the need for greater integration of routine screening for hereditary cancer risk factors for men in primary care settings to enhance early detection and preventive measures. Addressing testing barriers and promoting awareness among healthcare providers has the potential to improve cancer outcomes for male patients and their families.

## Introduction

Genetic screening has transformed the landscape of cancer prevention, offering patients and providers the power to uncover hidden hereditary risks and electively take action. Identifying pathogenic variants in hereditary cancer genes provides options for enhanced and targeted cancer surveillance, preventative interventions, and identifying at-risk relatives who would also benefit from testing, all of which can lower cancer morbidity and mortality (1, 2).

Increasing numbers of women are receiving genetic testing and benefitting from early cancer interventions (3–5). The United States Preventive Services Task Force (USPSTF) and American College of Obstetricians and Gynecologists (ACOG) recommend that primary care providers assess all women for risk of hereditary breast and ovarian cancer syndromes associated with *BRCA* variants. The USPSTF and the ACOG recommend offering genetic testing to women with elevated risk based on family history of cancer, personal history of cancer, and Ashkenazi Jewish ancestry (6, 7). ACOG has also published guidelines for when to offer testing for Lynch syndrome genes in patients with a personal history of endometrial or colon cancer, and in unaffected patients based on family and personal history (7). While there are multiple studies reporting recommendations and outcomes for hereditary cancer gene screening among women (8), there is a lack of published information on hereditary cancer testing in men (5, 9). This is particularly important given that men have higher lifetime rates of cancer and cancer mortality compared to women (10, 11).

Men are expected to have the same likelihood as women to carry pathogenic variants of hereditary cancer genes (12), and National Comprehensive Cancer Network (NCCN) guidelines support offering hereditary cancer testing to male patients based on family and personal cancer history (12–18). In a recent systematic review, Cheng et al. compared society guidelines for cancer screening and treatment for male patients found to be *BRCA1/2* pathogenic variant carriers (9). The review identified well-defined clinical management guidelines for men with *BRCA1/2* gene variants and the authors concluded that increasing identification of men with pathogenic cancer gene variants could reduce the burden of hereditary cancer via regular screening, early intervention, and education regarding familial risk (9). Despite these benefits, data from the 2015 US National Health Interview Survey estimates that men received hereditary cancer testing at one-tenth the frequency of women (5). Understanding of hereditary cancer genes and access to genetic testing have grown rapidly over the past decade, but there is a limited understanding of testing uptake and patient characteristics for men who meet guidelines for hereditary cancer testing.

Our objective was to address this gap by evaluating men undergoing genetic testing for hereditary cancer genes at a commercial laboratory and comparing the yield of tests, test results, and patient characteristics with women undergoing testing at the same laboratory. We hypothesized that men would have fewer test orders but higher rates of positive test results compared to women.

## Methods

### Study population

This retrospective analysis included men and women over the age of 18 years who received hereditary cancer testing from a single commercial laboratory between June 2020 and August 2023. Tests for individuals under the age of 18 years, international samples, and samples that did not produce a result were excluded from the analysis. For patients who had more than one sample submitted during the study period, the results from the largest panel of genes ordered were included in the analysis. Patient characteristics (biological sex, age, family and personal history of cancer, type of personal cancer, and race and ethnicity) and ordering clinic type (family/internal medicine; OB/GYN/women’s health; or specialty clinic including genetic counseling, cancer screening centers, surgery centers, or others) were obtained from the test requisition form. This study was granted a waiver of the consent process and for documentation of informed consent under 45 CFR 46.116(c)(d) under the local IRB determination (Salus IRB, ID# 21204-01A).

### Hereditary cancer testing

Hereditary cancer gene testing was performed using next-generation sequencing (NGS) based multiplex gene panels (Empower™, Natera, Inc. in collaboration with Baylor Genetics). Read depth analysis was used to detect copy number variation (CNV) for genes in the panel. Positive sequencing results were confirmed by gene-specific long-range PCR, Sanger sequencing, or multiplex ligation-dependent probe amplification (MLPA) (19). Variants detected in coding exons and within 20 base pairs of the exon/intron boundary were reported unless otherwise specified. Test results were categorized as pathogenic/likely pathogenic (P/LP) or negative. Variants of uncertain significance (VUS) were grouped with negative results. Variant classification was in accordance with the recommended guidelines from the American College of Medical Genetics and Genomics and the Association for Molecular Pathology, as previously described (20).

Testing panels ranged from two to 191 genes associated with hereditary cancer. For the purpose of this study, the panels were categorized as small (<20 genes), medium (20–53 genes) and large (>53 genes). In addition to pre-curated panels, providers could order a custom panel from a library of 191 genes. The genes included on each panel are listed in **Supplementary Table 1**. Genes were defined as clinically actionable if they were associated with recommendations for cancer surveillance or interventions based on the NCCN guidelines for clinical management and cancer risk surveillance (13–18) or additional literature-driven evidence (21–26). See **Supplementary Table 2** for details.

### Statistical analysis

Summary statistics of demographics, personal and family cancer histories, ordering clinic type, gene panel size, and the proportion of patients with positive genetic testing results (including the proportion of positive results that were considered clinically actionable) were calculated as counts and percentages. We also examined the genes that most frequently harbored a P/LP variant. To gain more insight into the context of testing, the type of clinic ordering the test was also examined. Differences in the distribution of binary variables between males and females were evaluated via odds ratios calculated from Fisher’s exact test. Differences in the distribution of continuous variables between males and females were evaluated using the Wilcoxon rank test. Point estimates and 95% confidence intervals (CI) were calculated for comparisons. Statistics were performed in R (versions 4.3.1 or above).

## Results

### Population characteristics

Of 234,005 tests performed during the study period, 9,964 were excluded based on study eligibility criteria (**Supplementary Figure 1**). Of the 224,041 individuals included in the analysis, 213,105 (95%) were female and 10,936 (5%) were male. Men were significantly older than women at the time of testing (54.2 *vs*. 43.4 years, p<0.001). Race and ethnicity data were available for 83% (186,294/224,041) of the study population, which was 47% (104,344/224,041) White, 14% (31,593/224,041) Hispanic, and 13% (28,498/224,041) Black. Compared to women, men who underwent testing for hereditary cancer genes were less likely to be Black (OR, 0.85, 95% CI, 0.80 to 0.91) and more likely to be of Ashkenazi Jewish ancestry (OR, 1.42, 95% CI, 1.23 to 1.62; **Table 1**).

**Table 1:** Patient characteristics.

Characteristics	Female n = 213,105	Male n = 10,936	p-value^a^ or odds ratio (95% CI)^b^
Median age in years (25%, 75%)	43.4 (34.2, 55.0)	54.2 (41.4, 65.9)	p<0.001
Race and ethnicity, n (%)			
White	99,306 (46.6%)	5,038 (46.1%)	0.98 (0.94 to 1.02)
Hispanic	30,093 (14.1%)	1,500 (13.7%)	0.97 (0.91 to 1.02)
Black	27,279 (12.8%)	1,219 (11.2%)	0.85 (0.80 to 0.91)
Ashkenazi Jewish	3,170 (1.5%)	229 (2.1%)	1.42 (1.23 to 1.62)
East Asian	2,625 (1.2%)	123 (1.1%)	0.91 (0.75 to 1.09)
Southeast Asian	941 (0.4%)	58 (0.5%)	1.20 (0.91 to 1.57)
South Asian	1,235 (0.6%)	77 (0.7%)	1.22 (0.95 to 1.53)
Multiracial or multiethnic	5,248 (2.5%)	247 (2.3%)	0.92 (0.80 to 1.04)
None of the above	7,416 (3.5%)	490 (4.5%)	1.30 (1.18 to 1.44)
Not recorded	35,792 (16.8%)	1,955 (17.9%)	1.08 (1.03 to 1.13)
Personal cancer history, n (%)			
Personal history	28,397 (13.3%)	3,001 (27.4%)	2.67 (2.55 to 2.79)
No personal history	34,893 (16.4%)	1,868 (17.1%)	0.37 (0.36 to 0.39)
Missing personal history information	149,815 (70.3%)	6,067 (55.5%)	1.13 (1.07 to 1.18)
Family cancer history, n (%)			
Family history	174,353 (81.8%)	7,874 (72.0%)	0.50 (0.48 to 0.53)
No family history	19,118 (9.0%)	1,721 (15.7%)	1.99 (1.89 to 2.10)
Missing family history information	19,634 (9.2%)	1,341 (12.3%)	1.38 (1.30 to 1.46)
Cascade tested,* n (%)			
	1,074 (0.5%)	355 (3.2%)	6.62 (5.85 to 7.49)

a Differences in distribution of continuous variables between males and females were evaluated via Wilcoxon rank test.

b Differences in distribution of binary variables between males and females were evaluated via odds ratios calculated from application Fisher’s exact test. Point estimates and 95% confidence intervals (CI) were calculated for comparisons.

*Patients were reported as cascade tested if they participated in a family testing program following a positive finding in a blood relatives tested at the same commercial lab as the study cohort member. Cohort members whose family members were tested at a different lab, or who didn’t participate in the study lab’s family testing program, were not included in this category.

Compared to women, men had increased odds of having a personal history of cancer (OR, 2.67, 95% CI, 2.55 to 2.79) and decreased odds of reporting a family history of cancer (OR, 0.50, 95% CI 0.48 to 0.53; **Table 1**). The odds of having missing data on family cancer history were also greater in men than women (OR, 1.38, 95% CI 1.30 to 1.46). Men had 6.62 times greater odds of having had genetic testing as a result of cascade testing (testing relatives of an individual with a known pathogenic variant) compared to women (95% CI, 5.85 to 7.49; **Table 1**).

Among men with a reported personal history of cancer (n=3,001), prostate cancer (33.3%; 1,000/3,001), colorectal cancer (29.2%; 876/3,001), and pancreatic cancer (9.2%; 276/3,001) were the most common (**Supplementary Table 3**). Among women with a personal history of cancer (n=28,397), breast cancer (66.1%; 18,768/28,397), colorectal cancer (6.2%; 1,751/28,397), and ovarian cancer (5.3%; 1,499/28,397) were the most commonly reported. Data regarding personal history of cancer were incomplete or missing for 55.5% (6,067/10,936) of men and 70.3% (149,815/213,105) of women (**Table 1**).

### Test orders and results

Most orders for hereditary cancer testing for men came from family/internal medicine clinics (38%), and most orders for women came from women’s health clinics (74%), many of which provide primary care for women. Compared to women, testing for men was more likely to have been ordered by specialty clinics (OR, 5.73, 95% CI 5.44 to 6.02) (**Table 2**). Over the study period (2020–2023), the proportion of tests ordered for men increased from 2.0% to 5.7% (**Supplementary Table 4**). The number of genes included in the tests also differed between men and women (**Table 3**). Men had 3.03 (95% CI, 2.91 to 3.17) and 4.35 (95% CI, 3.55 to 5.29) times the odds of having large or custom panels ordered, respectively, compared to women.

**Table 2:** Proportion of test orders by clinic type.

Clinic type	Female n = 213,105	Male n = 10,936	Odds ratio (95% CI)^a^
Family or internal medicine	14,060 (6.6%)	4,110 (37.6%)	8.52 (8.17, 8.89)
OB/GYN or women’s health	157,926 (74.1%)	1,628 (14.9%)	0.06 (0.06, 0.06)
Specialty or other clinics	41,119 (19.3%)	5,198 (47.5%)	3.79 (3.64, 3.94)

a Differences in distribution of binary variables between males and females were evaluated via odds ratios calculated from application Fisher’s exact test. Point estimates and 95% confidence intervals (CI) were calculated for comparisons.

**Table 3:** Hereditary cancer test characteristics.

Characteristics	Female n = 213,105	Male n = 10,936	Odds ratio (95% CI)^a^
Negative result, n (%)			
	195,327 (91.7%)	9,413 (86.1%)	0.56 (0.53, 0.60)
P/LP result, n (%)			
	17,778 (8.3%)	1,523 (13.9%)	1.78 (1.68, 1.88)
P/LP result in at least one clinically actionable gene, n (%)			
	12,597 (5.9%)	1,142 (10.4%)	1.86 (1.74, 1.98)
Orders based on gene panel size, n (%)			
Small (2–19 genes)	29,327 (13.8%)	645 (5.9%)	0.39 (0.36, 0.43)
Medium (40 or 53 genes)	155,673 (73.0%)	6,770 (61.9%)	0.60 (0.58, 0.62)
Large (81 genes)	27,535 (12.9%)	3,395 (31.0%)	3.03 (2.91, 3.17)
Custom	570 (0.3%)	126 (1.2%)	4.35 (3.55, 5.29)

a Differences in distribution of binary variables between males and females were evaluated via odds ratios calculated from application Fisher’s exact test. Point estimates and 95% confidence intervals were calculated for comparisons. P/LP, pathogenic or likely pathogenic variant.

P/LP variants were reported in 13.9% (1,523/10,936) of men compared to only 8.3% (17,778/213,105) of women (OR, 1.78, 95% CI, 1.68 to 1.88). When stratified by panel size, men had higher rates of P/LP variants for all panels except for a 5-gene Lynch Syndrome panel (**Supplementary Table 5**). Further, over the study period, the prevalence of P/LP variants observed in men increased from 10.7% to 15.0%, although this change was not statistically significant (**Supplementary Table 4**).

**Figure 1:**
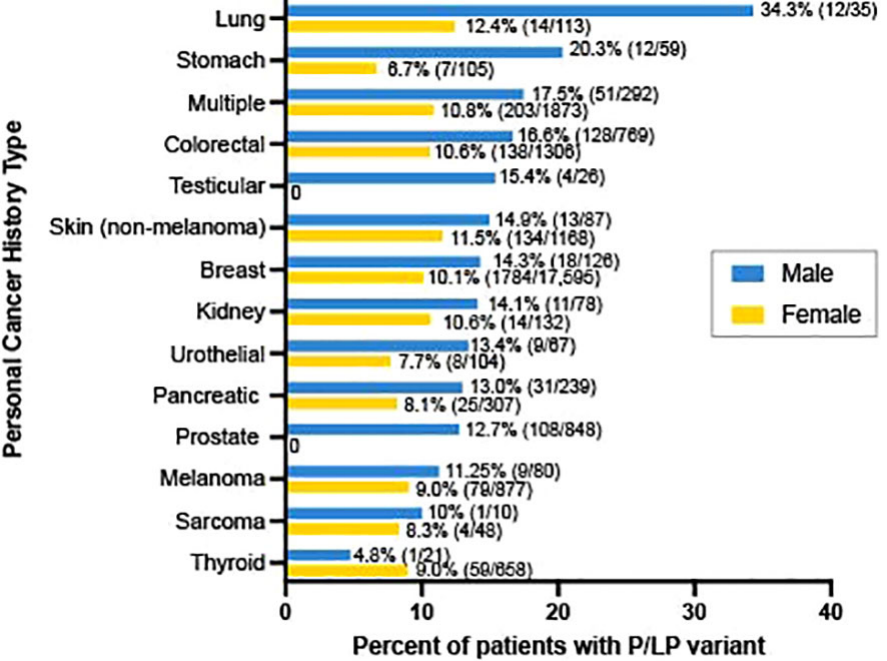
Percent of patients with a P/LP variant by personal history of cancer type. Among patients with known cancer type in personal history, the percent of men (blue) and women (yellow) with a pathogenic or likely pathogenic variant are shown. Excludes patients with a personal history of cancer where the cancer type was not specified (n=125 in men; n=869 in women), and cancer types observed in fewer than 5 men (small bowel, n=4; brain, n=2; endometrial, n=0; and ovarian, n=0).

Among patients with a P/LP variant, a similar proportion of men and women had data available on personal cancer history: 84.4% (1,285/1,523) among men and 84.3% (14,981/17,778) among women. Of those, 35.1% (451/1,285) of men with a P/LP variant reported a personal history of cancer compared to 19.3% (2,892/14,981) of women. The cancer types most frequently associated with a P/LP finding in men were lung (34.3% [12/35] of men with personal history of lung cancer had a P/LP variant), stomach (20.3%; 12/59), multiple cancer types (17.5%; 51/292), and colorectal cancer (16.6%; 128/769) (**Figure 1**).

Finally, men had greater odds of having a P/LP variant in at least one clinically actionable gene (OR, 1.86, 95% CI 1.74 to 1.98) (**Table 3**). *BRCA1/2* variants were the most frequently identified actionable variants and were more likely to be found in men than women (OR, 3.16, 95% CI, 2.70 to 3.67). Men also had greater odds of testing positive for *CHEK2, ATM*, Lynch syndrome-associated genes, and *APC* variants (**Table 4**).

**Table 4:** Genes most frequently harboring pathogenic or likely pathogenic variants.

Genes	Female n/N* (%)	Male n/N* (%)	Odds Ratio (95% CI)^a^
*BRCA1/2*	3777/212,774 (1.8%)	375/10,801 (3.5%)	3.16 (2.70, 3.67)
*CHEK2*	2695/198,900 (1.4%)	176/10,347 (1.7%)	1.26 (1.07, 1.47)
*ATM*	1271/198,899 (0.6%)	113/10,347 (1.1%)	1.72 (1.40, 2.08)
Lynch syndrome genes *(MLH1, MSH2, MSH6, PMS2, EPCAM)*	1240/199,147 (0.6%)	203/10,465 (1.9%)	1.99 (1.78, 2.22)
*APC*	821/183,657 (0.5%)	90/10,269 (0.9%)	1.97 (1.56, 2.45)

*n=Number of pathogenic or likely pathogenic variants detected; N=total number of patients tested.

a Differences in distribution of binary variables between males and females were evaluated via odds ratios calculated from application Fisher’s exact test. Point estimates and 95% confidence intervals (CI) were calculated for comparisons.

## Discussion

There is a limited understanding of uptake and characteristics among men undergoing hereditary cancer genetic testing. This study aimed to address this gap by evaluating the yield of hereditary cancer genetic testing and clinical characteristics among men compared to women in a large commercial laboratory sample cohort in the US. In our study cohort, only 5% of genetic tests were from male patients and the median age at testing was approximately 11 years older than that of women. A personal history of cancer was reported in 27% of men compared to 13% of women. While the highest proportion of test orders for men and women were primary care and women’s health clinics, respectively, men were more likely to have tests ordered from specialty clinics. Further, men were more likely than women to have a positive result. These findings may reflect that men receiving hereditary cancer testing are more likely to be tested in the setting of active cancer care for confirmatory germline genetic testing following tumor-variant testing, or treatment decision-making via genetics-informed care such as PARP inhibitors for prostate cancer patients with *BRCA1/2* variants (9).

The overall prevalence of P/LP variants reported in this study (13.9% of men and 8.3% had a P/LP variant) is consistent with the real-world mixture of clinical experiences in our cohort, which included patients with and without a personal history of cancer. For comparison, recent estimates of the P/LP variant rate in cancer susceptibility genes in an unselected, general population was 5% compared to another study reporting rates of 10–30% of patients with cancer (27, 28). Of clinical importance is that 10.4% of men in our cohort had P/LP variants that were actionable (*vs* 5.9% of women) and warranted a change in clinical management. These results suggest that there are differences in testing pathways for men and there may be opportunities for expanding hereditary cancer genetic testing access and uptake among men, including those without a personal cancer diagnosis.

There are several benefits associated with genetic cancer testing, and there are validated screening and treatment options for men with pathogenic hereditary cancer gene variants (9). However, studies suggest that men face barriers to receiving genetic counseling for hereditary cancer (5, 29). This is likely attributable to sociodemographic, psychosocial, and clinical factors (29), such as lower health-seeking behaviors and preventive care attendance compared to women, which may create differences in referral pathways (11). Women have routine, repeated encounters with health care providers via gynecological and obstetric care, which increases opportunities for referral and counseling. Moreover, U.S. national survey statistics show that men are less likely to visit community-based clinics (30), have a physician visit (31), or have health insurance coverage (32). These disparities may be associated with a higher threshold among men for seeking specialist care, and a greater need for tangible risk factors (e.g., personal experience with cancer) to accept hereditary cancer testing.

There is also a lack of male-focused hereditary cancer screening guidelines from an organization equivalent to those of ACOG for women and fewer male-focused cancer advocacy groups. This could contribute to a gap in screening for hereditary risk factors and offering hereditary testing, which is supported by the data showing that testing seems to be more conservative with men being older and more likely to have a positive personal history of cancer at the time of testing than women. Men also had a higher yield for P/LP and specifically actionable gene variants.

Family history of cancer plays a key role in providers’ decisions to offer genetic cancer testing. However, previous research has found that men have significantly lower availability and comprehension of cancer family history (33). Consistent with this, fewer men in our cohort reported a family history of cancer and a larger proportion of men had missing data regarding family history of cancer. These results highlight the importance of eliciting accurate health history in the context of offering men genetic cancer testing.

Given growing indications for hereditary cancer genetic testing (2), there is an increasing need for primary care providers to understand and integrate cancer genetic evaluations for men into their practices (34). The similar rates of orders arising from primary care and specialty clinics and the higher rate of a personal history of cancer relative to women suggests that gene cancer screening guidelines for men in the primary care setting could lead to earlier and more robust uptake of cancer genetic testing. Existing data on primary care and genetic testing demonstrates that primary care providers have an incomplete understanding of cancer gene testing and often express concerns about the ethical, legal, and social implications of testing (35). Further, existing research does not prioritize understanding male-specific hereditary cancer testing in the primary care setting. Giri et al. proposed an integrated model for collaboration between primary care and genetic counselors for the genetic evaluation of prostate cancer (34); however, more work is needed to expand these collaborative models to other cancers that affect men and integrate these into the primary care setting. Implementing standardized hereditary cancer screening protocols in primary care may promote a more equitable uptake of early hereditary cancer screening.

**Figure 2:**
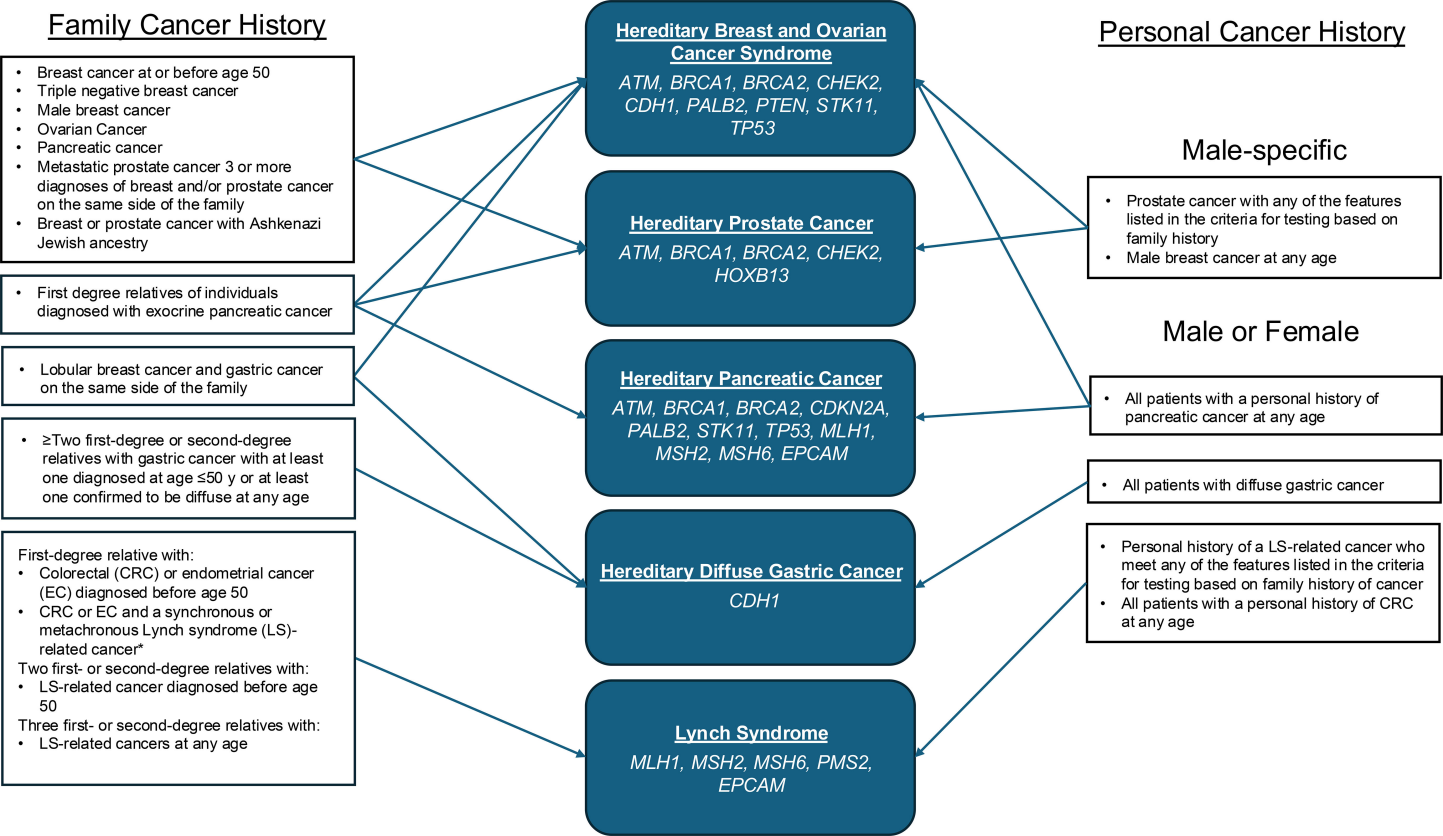
Hereditary cancer genetic testing recommendations. Recommendations for hereditary cancer syndromes (and associated genes) to test for based on family cancer history (left) and personal cancer history stratified by cancers specific to male patients and cancers applicable to both male and female patients (right). All information presented is summarized from the National Comprehensive Cancer Network guidelines available at the time of the study (13–18).

Though not specific to primary care, the NCCN and other professional societies outline which genes to test for based on personal and family cancer history. Testing for *BRCA1/2* variants is recommended for those at risk of hereditary prostate and male breast cancer, along with other cancer types that are not specific to men (**Figure 2**) (13–18). Men in our cohort had a significantly higher likelihood of *BRCA1/2* variants than women. Combined with the increased burden of cancer among men in this population, these findings align with previous research demonstrating a higher likelihood of *BRCA1/2* variants in men with a personal cancer history (28, 36). *BRCA* variant risk prediction tools exist to best advise patients regarding hereditary cancer genetic testing along with education regarding family cancer risk; these tools have been optimized for women with breast and ovarian cancer, but have been found unreliable among men with prostate cancer (37). In addition, prior work suggests that genetic counseling for men with pathogenic variants in *BRCA1/2* may be under-offered or under-utilized (38). Though men with *BRCA 1/2* variants are at a relatively lower risk of developing cancer compared to women, there are still important health implications for the individuals and their female relatives (e.g., personal risk of developing prostate cancer or breast cancer and the risk of ovarian and breast cancer for daughters) (39). Optimizing prediction tools and genetic counseling for men with *BRCA* variants may support earlier identification of pathogenic cancer gene variants and cancer risk management.

The NCCN provides hereditary cancer genetic testing guidelines for male patients across several cancer types. NCCN is widely referenced in oncology clinics but may not be routinely used in the primary care setting. ACOG and USPSTF have guidelines related to screening and testing for hereditary cancer among women but there is not an equivalent set of guidelines for male patients that is universally used in the primary care setting. In 2024, the American Society for Clinical Oncologists published guidelines for hereditary cancer syndrome testing in patients with cancer (40). These guidelines included the recommendation that all cancer patients have a family history taken and recorded and that germline genetic testing be offered when indicated. Greater awareness of the importance of hereditary cancer screening in cancer patients may improve testing rates among affected men. However, to promote informed decision-making regarding hereditary cancer screening among all men, future research and targeted efforts are needed to increase the accurate identification of family cancer history among men, improve hereditary cancer risk prediction tools specific to male patients, and address low rates of men receiving genetic counseling and preventative care. Consistently screening male patients for hereditary cancer and offering genetic testing will improve early cancer detection rates and cancer prevention for both the patient and, potentially, relatives. Finally, primary care providers are an important access point for men and greater attention to hereditary cancer screening in primary care settings could be bolstered by the development of primary care-specific hereditary cancer testing guidelines for men.

This study analyzed a large sample of hereditary cancer tests ordered across the US, mostly from unaffected individuals. However, there are limitations that may affect the overall interpretation of the results. The data collected for this study was based entirely on information provided on the clinics’ test requisition forms, introducing potential bias due to reliance on patient knowledge/recall for family cancer history, or providers omitting details. This may in part explain the large proportion of patients with missing information on personal cancer history. Our findings may also be impacted by selection bias due to most test orders originating from OB/GYN or women’s health clinics, and our male cohort being older than our female cohort, which could enrich our male population with affected individuals due to the nature of cancer risk increasing with age. However, this still highlights the importance of offering hereditary cancer testing to healthy individuals to focus on prevention. In addition, the data was limited to ordering from a single commercial lab and may not fully reflect population-level genetic testing patterns. This may have affected several findings, including the rate of cascade testing, which would not include family members tested at another lab. Finally, NCCN guidelines for hereditary cancer testing are updated frequently and current guidelines may differ from those in use at the time of the study.

In conclusion, the real-world experience of a genetic testing lab revealed that male patients comprised a substantially smaller proportion of hereditary cancer test orders than women. Given that men have a higher burden of cancer than women and could benefit from early hereditary cancer screening, hereditary cancer genetic testing guidelines targeted to male patients in the primary care setting are needed and could increase testing uptake. Further research is needed to develop targeted interventions to reduce barriers that limit male patients’ access to hereditary cancer genetic testing. 

## Data Availability

Data will be available upon reasonable request from the corresponding author, except for primary sequencing data and other individual patient information. The specific variant level data are contributed to ClinVar by Baylor Genetics, Houston, Texas.
