# Supplementary material for: Testing for hereditary cancer genes in men: a missed opportunity for cancer prevention
Source: Front Oncol. 2026 Mar 16;16:1766711. doi: 10.3389/fonc.2026.1766711 (PMC13033552; doi:10.3389/fonc.2026.1766711)

**Supplementary Table 1.** Genes reported

| **Panel Name** | **Genes Included** |
| --- | --- |
| HBOC | *BRCA1, BRCA2* |
| Lynch syndrome | *EPCAM, MLH1, MSH2, MSH6, PMS2* |
| GYN-Guidelines Based (19 genes) | *ATM, BRCA1, BRCA2, BRIP1, CDH1, CHEK2, EPCAM, MLH1, MSH2, MSH6, NBN, NF1, PALB2, PMS2, PTEN, RAD51C, RAD51D, STK11, TP53* |
| Multi-Cancer Panel (40 genes) | *APC, ATM, AXIN2, BAP1, BARD1, BMPR1A, BRCA1, BRCA2, BRIP1, CDH1, CDK4, CDKN2A, CHEK2, EPCAM, GALNT12, GREM1, HOXB13, MEN1, MITF, MLH1, MSH2, MSH3, MSH6, MUTYH, NBN, NF1, NTHL1, PALB2, PMS2, POLD1, POLE, PTEN, RAD51C, RAD51D, RNF43, RPS20, SMAD4, STK11, TP53, VHL* |
| Comprehensive Panel (81 genes) | *AIP, ALK, APC, ATM, AXIN2, BAP1, BARD1, BMPR1A, BRCA1, BRCA2, BRIP1, CDC73, CDH1, CDK4, CDKN1B, CDKN1C, CDKN2A, CEBPA, CHEK2, CYLD, DDX41, DICER1, EGFR, EPCAM, EXT1, EXT2, FH, FLCN, GATA2, GREM1, HOXB13, KIT, LZTR1, MAX, MEN1, MET, MITF, MLH1, MSH2, MSH3, MSH6, MUTYH, NBN, NF1, NF2, NTHL1, PALB2, PDGFRA, PHOX2B, PMS2, POLD1, POLE, POT1, PRKAR1A, PTCH1, PTEN, RAD51C, RAD51D, RB1, RET, RHBDF2, RUNX1, SDHA, SDHAF2, SDHB, SDHC, SDHD, SMAD4, SMARCA4, SMARCB1, SMARCE1, STK11, SUFU, TERC, TERT, TMEM127, TP53, TSC1, TSC2, VHL, WT1* |
| Multi-Cancer Expanded (53 genes) | *APC, ATM, AXIN2, BAP1, BARD1, BMPR1A, BRCA1, BRCA2, BRIP1, CDH1, CDK4, CDKN2A, CHEK2, CTNNA1, DICER1, EPCAM, GALNT12, GREM1, HOXB13, KIT, MEN1, MITF, MLH1, MRE11, MSH2, MSH3, MSH6, MUTYH, NBN, NF1, NTHL1, PALB2, PDGFRA, PMS2, POLD1, POLE, PTEN, RAD50, RAD51C, RAD51D, RNF43, RPS20, SDHA, SDHB, SDHC, SDHD, SMAD4, SMARCA4, STK11, TP53, TSC1, TSC2, VHL* |
| Genes Offered on 53 gene panel that are not included on the 19, 40 or 81 gene panel | *CTNNA1, MRE11, RAD50* |
| Genes Offered on the 40 gene panel that are not offered on the 81 gene panel (offered on the 53 gene panel) | *GALNT12, RNF43, RPS20* |
| All 191 genes available to test through custom panel | *ABRAXAS1, ACD, AIP, AKT1, ALK, ANKRD26, AP2S1, APC, ATM, ATR, AXIN2, BAP1, BARD1, BLM, BMPR1A, BRCA1, BRCA2, BRIP1, BUB1B, CASR, CDC73, CDH1, CDK4, CDKN1B, CDKN1C, CDKN2A, CEBPA, CEP57, CFTR, CHEK2, CPA1, CTC1, CTNNA1, CTR9, CTRC, CYLD, DDB2, DDX41, DICER1, DIS3L2, DKC1, EGFR, EGLN1, ENG, EPCAM, ERBB2, ERCC1, ERCC2, ERCC3, ERCC4, ERCC5, EXT1, EXT2, EZH2, FAN1, FANCA, FANCB, FANCC, FANCD2, FANCE, FANCF, FANCG, FANCI, FANCL, FANCM, FBXW7, FH, FLCN, GALNT12, GATA1, GATA2, GEN1, GNA11, GPC3, GREM1, HNF1A, HNF1B, HOXB13, HRAS, KDM3B, KIF1B, KIT, LZTR1, MAX, MC1R, MDM2, MEN1, MET, MITF, MLH1, MLH3, MRE11, MSH2, MSH3, MSH6, MUTYH, NBN, NF1, NF2, NHP2, NOP10, NTHL1, NYNRIN, PALB2, PALLD, PARN, PAX5, PDGFRA, PHOX2B, PIK3CA, PMS1, PMS2, POLD1, POLE, POLH, POT1, PRF1, PRKAR1A, PRSS1, PTCH1, PTCH2, PTEN, RAD50, RAD51C, RAD51D, RB1, RECQL, RECQL4, REST, RET, RHBDF2, RINT1, RNF43, RPL11, RPL15, RPL18, RPL19, RPL26, RPL27, RPL35, RPL35A, RPL5, RPS10, RPS15A, RPS17, RPS19, RPS20, RPS24, RPS26, RPS27, RPS28, RPS29, RPS7, RTEL1, RUNX1, SAMD9L, SDHA, SDHAF2, SDHB, SDHC, SDHD, SLC45A2, SLX4, SMAD4, SMARCA4, SMARCB1, SMARCE1, SPINK1, SPRED1, SRP72, STK11, SUFU, TERC, TERT, TINF2, TMEM127, TP53, TRIM28, TRIP13, TSC1, TSC2, TSR2, TYR, USB1, VHL, WRAP53, WRN, WT1, XPA, XPC, XRCC2* |

# **Supplementary Table 2.** Clinical actionability of genes with pathogenic or likely pathogenic variants in the cohort per NCCN or other guidance.

| **Gene** | **Clinically actionable for males** | **Clinically actionable for females** |
| --- | --- | --- |
| *AIP* | No | No |
| *APC* | Yes^1^ | Yes^1^ |
| *ATM* | Yes^2^ | Yes^2^ |
| *AXIN2* | Yes^1^ | Yes^1^ |
| *BAP1* | Yes^3^ | Yes^3^ |
| *BARD1* | No | Yes^2^ |
| *BLM*: biallelic | Yes^4^ | Yes^4^ |
| *BLM:* monoallelic | No | No |
| *BMPR1A* | Yes^1^ | Yes^1^ |
| *BRCA1* | Yes^2^ | Yes^2^ |
| *BRCA2* | Yes^2^ | Yes^2^ |
| *BRIP1* | No | Yes^2^ |
| *CDC73* | No | No |
| *CDH1* | Yes^2^ | Yes^2^ |
| *CDKN1B* | No | No |
| *CDKN1C* | Yes^5^ | Yes^5^ |
| *CDKN2A* | Yes^2^ | Yes^2^ |
| *CFTR:* monoallelic | No | No |
| *CHEK2* | Yes^2^ | Yes^2^ |
| *CTRC* | No | No |
| *CYLD* | No | No |
| *DDX41* | Yes^6^ | Yes^6^ |
| *DICER1* | Yes^7^ | Yes^7^ |
| *EGFR* | No | No |
| *EPCAM* | Yes^1^ | Yes^1^ |
| *ERCC2:* monoallelic | No | No |
| *ERCC3:* monoallelic | No | No |
| *EXT1* | No | No |
| *EXT2* | No | No |
| *FANCA:* monoallelic | No | No |
| *FANCM:* monoallelic | No | No |
| *FH* | Yes^3^ | Yes^3^ |
| *FLCN* | Yes^3^ | Yes^3^ |
| *GREM1* | Yes^1^ | Yes^1^ |
| *HOXB13* | Yes^2,8^ | No |
| *LZTR1* | Yes^9^ | Yes^9^ |
| *MEN1* | Yes^10^ | Yes^10^ |
| *MITF* | No | No |
| *MLH1* | Yes^1^ | Yes^1^ |
| *MRE11:* monoallelic | No | No |
| *MSH2* | Yes^1^ | Yes^1^ |
| *MSH3*: biallelic | Yes^1^ | Yes^1^ |
| *MSH3:* monoallelic | No | No |
| *MSH6* | Yes^1^ | Yes^1^ |
| *MUTYH*: biallelic | Yes^1^ | Yes^1^ |
| *MUTYH:* monoallelic | No | No |
| *NBN*: biallelic | No | No |
| *NBN:* monoallelic | No | No |
| *NF1* | Yes^2^ | Yes^2^ |
| *NF2* | Yes^9^ | Yes^9^ |
| *NTHL1*: biallelic | Yes^1^ | Yes^1^ |
| *NTHL1:* monoallelic | No | No |
| *PALB2* | Yes^2^ | Yes^2^ |
| *PHOX2B* | No | No |
| *PMS2* | Yes^1^ | Yes^1^ |
| *POLD1* | Yes^1^ | Yes^1^ |
| *POT1* | No | No |
| *PRF1* | No | No |
| *PRKAR1A* | No | No |
| *PTEN* | Yes^2^ | Yes^2^ |
| *RAD50* | No | No |
| *RAD51C* | No | Yes^2^ |
| *RAD51D* | No | Yes^2^ |
| *RECQL4:* monoallelic | No | No |
| *RET* | Yes^10^ | Yes^10^ |
| *RTEL1:* monoallelic | Yes^6^ | Yes^6^ |
| *RUNX1* | Yes^6^ | Yes^6^ |
| *SDHA* | Yes^3,10^ | Yes^3,10^ |
| *SDHAF2* | Yes^3,10^ | Yes^3,10^ |
| *SDHB* | Yes^3,10^ | Yes^3,10^ |
| *SDHC* | Yes^3,10^ | Yes^3,10^ |
| *SDHD* | Yes^3,10^ | Yes^3,10^ |
| *SMAD4* | Yes^1^ | Yes^1^ |
| *SMARCA4* | Yes^11,12^ | Yes^11,12^ |
| *SMARCE1* | No | No |
| *SPINK1* | No | No |
| *STK11* | Yes^1^ | Yes^1^ |
| *SUFU*: biallelic | No | No |
| *SUFU:* monoallelic | Yes^11^ | Yes^11^ |
| *TERT*: biallelic | Yes^6^ | Yes^6^ |
| *TERT:* monoallelic | Yes^6^ | Yes^6^ |
| *TMEM127* | Yes^3,10^ | Yes^3,10^ |
| *TP53* | Yes^2^ | Yes^2^ |
| *TSC1* | Yes^3^ | Yes^3^ |
| *TSC2* | Yes^3^ | Yes^3^ |
| *TYR* | No | No |
| *VHL* | Yes^3^ | Yes^3^ |
| *WRAP53*: biallelic | No | No |
| *WRAP53*: monoallelic | No | No |
| *WT1* | Yes^13^ | Yes^13^ |

^1^Gupta S, Provenzale D, Llor X, et al. NCCN Guidelines Insights: Genetic/Familial High-Risk Assessment: Colorectal, Version 2.2019. J Natl Compr Canc Netw. Sep 1 2019;17(9):1032-1041.

^2^Daly MB, Pilarski R, Yurgelun MB, et al. NCCN Guidelines Insights: Genetic/Familial High-Risk Assessment: Breast, Ovarian, and Pancreatic, Version 1.2020. J Natl Compr Canc Netw. 2020;18(4):380-391.

^3^Motzer RJ, Jonasch E, Agarwal N, et al. Kidney Cancer, Version 3.2022, NCCN Clinical Practice Guidelines in Oncology. *J Natl Compr Canc Netw*. 2022;20(1):71-90.

^4^[Cunniff C, Djavid AR, Carrubba S, et al. Health supervision for people with Bloom syndrome. Am J Med Genet A. Sep 2018;176(9):1872-1881. doi:10.1002/ajmg.a.40374](https://web.endnote.com/reference-list/)

^5^Brioude F, Kalish JM, Mussa A, et al. Expert consensus document: Clinical and molecular diagnosis, screening and management of Beckwith-Wiedemann syndrome: an international consensus statement. Nat Rev Endocrinol. 2018;14(4):229-249.

^6^Greenberg PL, Stone RM, Al-Kali A, et al. NCCN Guidelines® Insights: Myelodysplastic Syndromes, Version 3.2022. J Natl Compr Canc Netw. 2022;20(2):106-117.

^7^Schultz KAP, Williams GM, Kamihara J, et al. DICER1 and Associated Conditions: Identification of At-risk Individuals and Recommended Surveillance Strategies. Clin Cancer Res. 2018 May 15;24(10):2251-2261. doi: 10.1158/1078-0432.CCR-17-3089. Epub 2018 Jan 17.

^8^Schaeffer EM, Srinivas S, Adra N, et al. Prostate Cancer, Version 4.2023, NCCN Clinical Practice Guidelines in Oncology. J Natl Compr Canc Netw. Oct 2023;21(10):1067-1096

^9^Evans DGR, Salvador H, Chang VY, et al. Cancer and Central Nervous System Tumor Surveillance in Pediatric Neurofibromatosis 2 and Related Disorders. *Clin Cancer Res*. 2017;23(12):e54-e61.

^10^Shah MH, Goldner WS, Benson AB, et al. Neuroendocrine and Adrenal Tumors, Version 2.2021, NCCN Clinical Practice Guidelines in Oncology. *J Natl Compr Canc Netw*. 2021;19(7):839-868. Published 2021 Jul 28.

^11^Foulkes WD, Kamihara J, Evans DGR, et al. Cancer Surveillance in Gorlin Syndrome and Rhabdoid Tumor Predisposition Syndrome. *Clin Cancer Res*. 2017;23(12):e62-e67.

^12^Frühwald MC, Nemes K, Boztug H, et al. Current recommendations for clinical surveillance and genetic testing in rhabdoid tumor predisposition: a report from the SIOPE Host Genome Working Group. *Fam Cancer*. 2021;20(4):305-316.

^13^Balis F, Green DM, Anderson C, et al. Wilms Tumor (Nephroblastoma), Version 2.2021, NCCN Clinical Practice Guidelines in Oncology. J Natl Compr Canc Netw. 2021;19(8):945-977. Published 2021 Aug 1.

# **Supplementary Table 3. Cancer types among patients with reported personal cancer history**

|  | **Female (N=28,397)** | | **Male (3,001)** | |
| --- | --- | --- | --- | --- |
| **Cancer Type** | **n** | **%** | **n** | **%** |
| Breast | 17595 | 62.0 | 126 | 4.2 |
| Ovarian | 1123 | 4.0 | 0 | 0.0 |
| Prostate | 0 | 0.0 | 848 | 28.3 |
| Pancreatic | 307 | 1.1 | 239 | 8.0 |
| Colorectal | 1306 | 4.6 | 769 | 25.6 |
| Endometrial | 1119 | 3.9 | 0 | 0.0 |
| Stomach | 105 | 0.4 | 59 | 2.0 |
| Urothelial | 104 | 0.4 | 67 | 2.2 |
| Small bowel | 10 | 0.0 | 4 | 0.1 |
| Brain | 32 | 0.1 | 2 | 0.1 |
| Kidney | 132 | 0.5 | 78 | 2.6 |
| Testicular | 0 | 0.0 | 26 | 0.9 |
| Thyroid | 658 | 2.3 | 21 | 0.7 |
| Lung | 113 | 0.4 | 35 | 1.2 |
| Skin, non melanoma | 1168 | 4.1 | 87 | 2.9 |
| Skin, melanoma | 877 | 3.1 | 80 | 2.7 |
| Sarcoma | 48 | 0.2 | 10 | 0.3 |
| Other | 958 | 3.4 | 133 | 4.4 |
| Multiple | 1873 | 6.6 | 292 | 9.7 |
| Not specified | 869 | 3.1 | 125 | 4.2 |

#

# **Supplementary Table 4. Proportion of tests ordered for male patients and positivity rate over the study period.**

| **Test year** | **Proportion of all tests ordered that were for men** | **Positivity rate for tests ordered for male patients** | **Positivity rate for tests ordered for female patients** |
| --- | --- | --- | --- |
| **2020** | 2.0% | 10.7% | 7.4% |
| **2021** | 4.1% | 11.9% | 7.9% |
| **2022** | 5.2% | 14.5% | 8.5% |
| **2023** | 5.7% | 15.0% | 8.7% |

# **Supplementary Table 5. Rate of pathogenic and likely pathogenic variants in men and women by gene panel size**

| **Panel type** | **No. men tested** | **No. men with P/LP variant** | **Male P/LP rate (%)** | **No. women tested** | **No. women with P/LP variant** | **Female P/LP rate (%)** |
| --- | --- | --- | --- | --- | --- | --- |
| 2-gene *BRCA1/2* panel | 453 | 59 | 13.02 | 13873 | 438 | 3.16 |
| 5-gene Lynch Syndrome panel | 112 | 11 | 9.82 | 279 | 30 | 10.75 |
| 19-gene panel | 80 | 14 | 17.5 | 15175 | 951 | 6.27 |
| 40-gene panel | 3911 | 469 | 11.99 | 91521 | 7388 | 8.07 |
| 53-gene panel | 2859 | 394 | 13.78 | 64152 | 5970 | 9.31 |
| 81-gene panel | 3395 | 558 | 16.44 | 27535 | 2896 | 10.52 |
| Custom size panel | 126 | 18 | 14.29 | 570 | 105 | 18.42 |

Abbreviation: P/LP, pathogenic or likely pathogenic

**Supplementary Figure 1.** **Flow diagram of samples excluded from analysis.**

The numbers of samples are presented in the order in which they were excluded (from top to bottom). Multiple tests indicate duplicate samples or multiple panels from the same patient, in which case only the largest panel was included.

Abbreviations TNP; test not performed


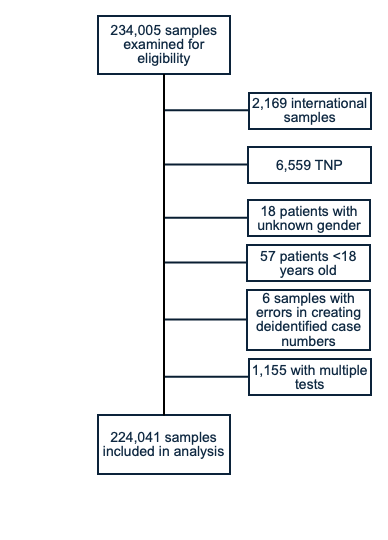

Supplement: Supplementary file 1 [file DataSheet1.docx]
